# Fine-needle aspiration biopsy for breast lesions: a comparison between two devices for obtaining cytological samples

**DOI:** 10.1590/S1516-31802005000600004

**Published:** 2005-11-01

**Authors:** Ruffo Freitas, Marise Amaral Rebouças Moreira, Gustavo Antônio de Souza, Ellen Hardy, Regis Resende Paulinelli

**Keywords:** Breast neoplasms, Diagnosis, Cytology, Techniques, Biopsy, Neoplasias mamárias, Diagnóstico, Citologia, Técnicas, Biópsia

## Abstract

**CONTEXT AND OBJECTIVE:**

Fine-needle aspiration biopsy has been accepted worldwide for breast lesions. However, some questions remain, including the appropriateness of the puncture method. The objective of this work was to compare aspirates obtained by the auto-vacuum device and by the syringe pistol holder.

**DESIGN AND SETTING:**

Randomized trial for validation of diagnostic method, at Hospital das Clínicas da Universidade Federal de Goiás and Hospital Araújo Jorge, Goiânia.

**METHODS:**

351 patients presenting breast lumps underwent fine-needle aspiration biopsy, either with the auto-vacuum device or the syringe pistol holder. A single cytopathologist analyzed all of the cytology slides. The rates of insufficient material, cellularity, cell distortion and background hemorrhage were evaluated.

**RESULTS:**

The percentages of insufficient material were 16% and 22% (p = 0.18), for the auto-vacuum and pistol aspirates, respectively. Good cellularity was seen in 34% of auto-vacuum and 29% of pistol samples (p = 0.4). Cell distortion was seen in 31 and 26 cases, respectively (p = 0.7). Background hemorrhage occurred in 63 (35%) and 54 cases (31%) (p = 0.2), for auto-vacuum and pistol. The sensitivity was 88% and 86%; specificity 99% and 100%, positive predictive value 96% and 100%, negative predictive value 96% and 95% and total accuracy 76% and 75% for the auto-vacuum and pistol, respectively.

**CONCLUSION:**

The results obtained from the two fine-needle aspiration biopsy methods were equivalent. Therefore, the auto-vacuum device is a good option for obtaining aspirates for cytology.

## RESUMO

**CONTEXTO E OBJETIVO:**

A punção aspirativa por agulha fina (PAAF) tem sido aceita em todo o mundo como parte da investigação diagnóstica das lesões mamárias. Não obstante, permanece por ser definida qual a melhor técnica na obtenção dos aspirados. O objetivo deste trabalho é comparar os aspirados obtidos pelo dispositivo de autovácuo com aqueles obtidos com a pistola de apreensão de seringa.

**TIPO DE ESTUDO E LOCAL:**

Estudo randomizado para validação de teste diagnóstico realizado no Departamento de Ginecologia e Obstetrícia da Universidade Federal de Goiás e Hospital Araújo Jorge da Associação de Combate ao Câncer em Goiás.

**MÉTODOS:**

Estudo aleatório, incluindo 351 pacientes, que apresentaram nódulos mamários e foram submetidas à PAAF, por um dos dois métodos de punção. Uma única citologista analisou todas as lâminas. As taxas de material insuficiente, celularidade, distorção celular e hemorragia de fundo foram analisados.

**RESULTADOS:**

A taxa percentual de material insuficiente foi de 16% e 2% (p = 0,18), para o dispositivo de autovácuo e para a pistola, respectivamente. Boa celularidade foi observada em 34% das amostras obtidas com o auto-vácuo e em 29% daquelas obtidas com a pistola (p = 0,4). Distorção celular foi vista em 31 casos e 26 casos, respectivamente (p = 0,7). Hemorragia de fundo ocorreu em 63 casos (35%) e em 54 (31%) (p = 0,2), para o autovácuo e para pistola. A sensibilidade foi de 88% e 86%; a especificidade de 99% e 100%, o valor preditivo positivo de 96% e 100%, o valor preditivo negativo de 96% e 95% e a acurácia total de 76% e 75% para o autovácuo e para a pistola, respectivamente.

**CONCLUSÃO:**

Os aspirados obtidos por ambas as técnicas foram equivalentes. Assim, o autovácuo se firma como uma boa opção na obtenção de aspirados para estudo citológico de lesões mamárias.

## INTRODUCTION

Fine-needle aspiration biopsy (FNAB) has been used throughout the world as a diagnostic tool for breast tumors. However, despite being a simple and practical procedure, the insertion technique has some peculiarities that must be taken into consideration.^1^

Because of its motility, the operator must keep the lump in a single position with one hand. Thus, only the other hand will be free to hold the syringe, pull the plunger (to generate a vacuum inside the syringe) and make the forward and backward movements needed for locating and puncturing the tumor. The three maneuvers are simple; however, when performed at the same time, uneven results can ensue, as sometimes the volume of the aspirate can be greater or smaller. In addition, the pulling movement exerted on the plunger can occasionally distract the operator, thereby causing a change in needle direction. These difficulties may make this method less than precise.^1,2^

In order to correct such difficulties, several health units have been using a pistol-grip mechanical syringe holder, first developed by Franzen and later modified by several manufacturing companies.^3^ This pistol-grip mechanical syringe holder makes fine-needle aspiration biopsy much easier to perform. However, the use of the holder causes the distance between the tumor and the hand holding the device to increase. This decreases the sensitivity of the operator's touch^4^ and, moreover, the size of the assembled device frequently frightens patients, who may become less willing to collaborate in the procedure.^5^

**Figure 1:**
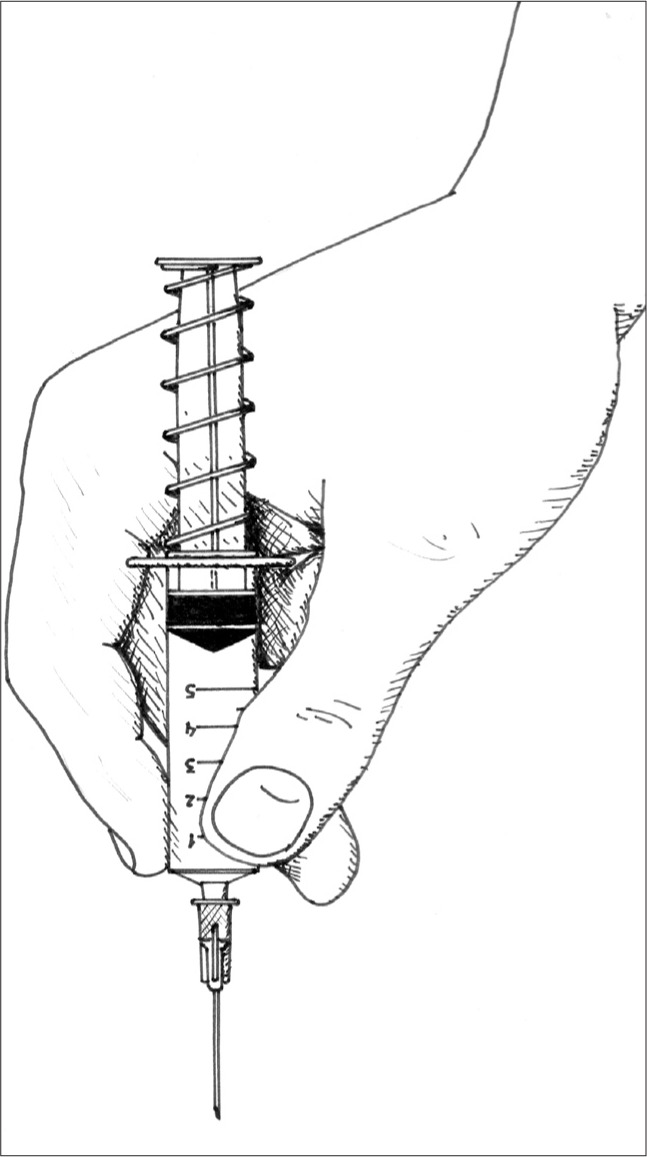
Fine-needle aspiration biopsy being performed using the auto-vacuum device.

The present authors have developed a different device with the purpose of facilitating the procedure and not frightening the patient. The idea consists of the interposition of a spring between the lateral wings of a disposable syringe and the plunger, so that the spring can exert a force on the plunger to cause distal traction, thus creating the required negative pressure inside the syringe. This vacuum would then be responsible for suctioning the collected material into the hollow needle. The operator no longer needs to actually create the vacuum, as the device does it by itself (Figure 1). This device has therefore been named the “auto-vacuum device”.^6^

Initially, the physical characteristics of a steel spring that would fit into a 5-ml syringe were defined at the School of Mechanical Engineering of Universidade Estadual de Campinas. An experimental study using rats brought about very encouraging results, since we were able to get good quality aspirates with every procedure, both for hard tumors (Walker) and for soft ones (SP1).^6^

Despite the good results obtained in the tests, the auto-vacuum device had not yet been compared either with the pistol-grip mechanical holder for syringes or with other FNAB techniques, in female breast tumors. The lack of conclusive evidence motivated us to find out which would be the best choice of device for fine-needle aspiration biopsy.

Thus, the objective of this study was to compare the aspirates generated by the auto-vacuum device and those obtained using the pistol-grip mechanical holder for syringes, in oncological cytology, by observing the following factors: rate of unsatisfactory aspirates, cellularity of the aspirates, background hemorrhage of the smear and cell distortion.

## METHODS

A randomized study was conducted to validate a diagnostic test, by comparing two fine-needle aspiration biopsy techniques for the diagnosis of solid breast tumors.

Prior approval for the study was given by the Ethics Committee of Hospital das Clínicas of the Universidade Federal de Goiás. Subsequently, 351 patients with solid breast nodules, who were going to undergo fine-needle aspiration biopsy as part of the diagnostic investigation, were recruited for the study. These women were invited to participate as volunteers. For that purpose, they gave their signed informed consent. Once they had been included in the study, the patients were randomly allocated to one of the two branches of the study. The use of neoadjuvant chemotherapy and/or radiotherapy were considered to be exclusion criteria.

### Fine-needle aspiration biopsy with the auto-vacuum device

The **material** consisted of a 25 × 7 mm disposable hypodermic needle; disposable 5-ml syringe; 40 mm-long steel spring with wire of diameter 1 mm, external diameter of 15 mm, compression capacity of 600 g/cm and final force of 1.5 kg/f (Figure 1), and two glass slides. **Technique:** After the site was thoroughly cleaned using a piece of cotton wool soaked in alcohol, a puncture was made directly over the tumor, with the non-dominant hand stabilizing the lesion, while the dominant hand manipulated the device. The spring was compressed between the plunger and the syringe, until the tip of the needle had punctured the skin. After the needle had penetrated, the fingers were withdrawn, thus releasing the tension in the spring, and allowing it to retract. As a consequence, a constant negative pressure was created inside the syringe. While gently holding the syringe, the operator made forward and backward movements, in order to reach several regions of the tumor. Concomitant rotation movements were also made.

After completing 10 to 15 movements, or when the presence of material in the bulb of the needle was verified, the movements were stopped. The needle was then disconnected from the syringe, so that the negative pressure inside the syringe would be cancelled, thus preventing the material inside the needle from inadvertently entering the syringe. Immediately after this, the needle was reconnected to the syringe and the whole device was withdrawn from the lesion.

The next step was to deposit all the harvested material onto glass slides (at least two) by pushing on the plunger abruptly. Once the aspirate was on the glass slide, a smear was made, and the material was spread with the aid of a clean slide placed at a 45° angle, in such a way as to make the smear as thin as possible. The glass slides with the smears were immediately fixed in 70% alcohol.

### Fine-needle aspiration biopsy with a pistol-grip mechanical syringe holder

The **material** consisted of a pistol-grip mechanical syringe holder, 20 ml disposable plastic syringe, 25 × 7 mm disposable hypodermic needle and two glass slides. **Technique:** After the nodule was stabilized in one position with the non-dominant hand, the needle of the device, which was held by the dominant hand, penetrated the skin. The pistol trigger was then pushed, causing consequent traction on the plunger, and creating negative pressure inside the syringe. In an attempt to keep the aspiration as constant as possible, the operator would make forward and backward movements combined with simultaneous rotation movements, throughout the region of the tumor.

The test was stopped after 10 to 15 forward and backward movements, or when it was possible to see harvested material in the bulb of the needle. The traction on the pistol was relaxed, so as to interrupt the aspiration, thus canceling the negative pressure inside the syringe and preventing material from being aspirated into the syringe. The device was then withdrawn from the tumor, and only then was the needle disconnected from the syringe. The syringe, without the needle, was then taken out of the holder, with a recoiled plunger, and the needle was then reconnected to the syringe. The plunger was then pushed, depositing the harvested material onto the glass slides. The smear was produced and fixed in the same manner as described for the previous method.

### Cytology

The glass slides for each patient were stained according to the Papanicolaou method.^7^ Cytological analysis was done with the aid of an optical microscope, by a single cytologist from the Department of Pathology of the University Hospital of Universidade Federal de Goiás.

The cytologist (M.A.R.M.) was not informed of the type of device that had been used for the harvest. During the test, the cytologist would fill in the fields of the cytological report, as fully as possible, describing the diagnosis, cellularity, presence or absence of excessive background quantities of red blood cells, and the presence or absence of cell distortion, according to the variables that had been previously defined.

### Definition of variables

The **patient's age** was recorded in complete years, at the time she entered the study.

The **tumor size** was measured at its largest diameter, using calipers, on the day the puncture was performed. The **clinical stage** of the tumor was defined in accordance with the TNM classification of the Union Internationale Contre le Cancer.^8^ The **histological classification** of the tumors was done in accordance with guidelines laid down by the World Health Organization.^9^

**Cytological diagnosis:** the tumor was considered “malignant” when there was the presence of several isolated cells or cell aggregates with a hyperchromatic nucleus, prominent nucleolus and lack of proportion between nucleus and cytoplasm. The criteria for classifying a tumor as “suspicious” included the presence of cells whose cytomorphological features were similar to those of malignant tumors, but whose count was insufficient for a definite diagnosis. “Benign” tumors consisted of material composed of cells that did not present any cytomorphological changes. “Insufficient material” was the classification given to the slides that did not contain any cells.^10^ The classification of “abundant” **cellularity** was given to material with 15 or more cell blocks; “moderate” indicated the presence of five to 14 cell blocks and the cellularity was considered “scarce” when less than five blocks were present.

**Hemorrhagic background:** the cytological analysis was considered impaired when the number of red blood cells was so intense that it interfered with the cytological findings. “Present, but not interfering with the result” was the classification used in the cases where, in spite of the large quantities, the red blood cells did not interfere with the identification of the other cell types. “Absent” was used in the cases where no red blood cells were found, or where they were present in minute quantities.

**Cell distortion:** was reported as “present” when cell elongation, destruction or similar features could be seen by the cytologist, and “absent” when no distortion could be seen.

### Statistical analysis

The chi-squared test was used to compare the characteristics of the samples obtained using the two puncture methods. However, the means of the quantitative variables were compared via Student's t test for independent samples.^11^ The chi-squared test was used to analyze the differences between the two groups, with regard to cellularity, hemorrhagic background and cell distortion.^11^ To analyze accuracy, the patients with suspicious or insufficient samples (nondiagnostic samples) were excluded. Thus, this analysis included 273 patients in total (135 in the auto-vacuum group and 138 in the pistol group). The histology of the specimen was considered to be the gold standard.^11^ The confidence level utilized was 95%, and results were considered to be statistically significant when the p value was less than 0.05.

## RESULTS

Of the 351 patients who underwent puncture, 180 (51.3%) were allocated to the group in which the auto-vacuum device was used, and the other 171 (48.7%) to the group in which the pistol-grip mechanical syringe holder was used. Two-thirds of the patients were recruited at the University Hospital of Universidade Federal de Goiás (70%) and the others at the Araújo Jorge Hospital.

The mean age of the women who took part in the study was 38 years, ranging from 12 to 82 years (standard deviation, SD: ± 14). The mean size of the tumors was 27 mm, ranging from 5 to 170 mm (SD: ± 20). In 68 cases (18%) the tumors measured up to 10 mm, in 136 (36%) they were between 11 and 20 mm and in 176 patients (46%), the nodule measured more than 20 mm.

Among the 351 cases in which puncture were performed, 295 patients (84%) had the lesion removed at a later time. The remaining 56 patients (16%) either did not have their lesions removed or the procedure was done in another center. Follow-up was done for more than one year for 30 women, out of the 85 patients who did not have a histological confirmation. Their lesions remained clinically unchanged and growth was arrested.

Among the 295 patients who underwent excision of the nodule, histology showed that in 91 (31%) cases, the neoplasias were malignant, while in the remaining 204 women (69%) the lesions were benign. Table 1 shows the type of lesion detected through histology.

**Table 1.** Distribution of 295 excised tumors of the breast, according to histological findings

Histological type	n	%
Invasive ductal carcinoma	82	27.6
Invasive lobular carcinoma	4	1.3
Invasive cribriform carcinoma	1	0.4
Papilliferous carcinoma in situ	1	0.4
Malignant phyllodes tumor	1	0.4
Melanoma metastasis	1	0.4
Lymphoma	1	0.4
Fibroadenoma	132	44.6
Fibrocystic disorders	46	15.5
Lipoma	10	3.3
Radial scar	1	0.4
Adenoma	4	1.3
Chronic inflammatory process	3	1.0
Gynecomastia	2	0.7
Fatty necrosis	1	0.4
Lymphadenopathy	1	0.4
Hamartoma	1	0.4
Papilloma	2	0.7
Benign phyllodes tumor	1	0.4
**Total**	**295**	**100**

With the aim of certifying whether the groups were homogeneous and comparable, the cases that underwent puncture were distributed numerically and by percentage, within the various categories, according to the puncture method used. These numbers are presented in Table 2, from which it can be seen that there were no statistically significant differences for any of the control variables, thus showing that the groups were indeed homogeneous. For one of the patients who underwent puncture with the auto-vacuum device, the data regarding the staging of the tumor were missing.

**Table 2.** Distribution of 351 patients, according to age, tumor size and its clinical stage and the attending center, and the device used for the fine-needle aspiration biopsy of the breast: auto-vacuum or pistol-grip

Variable	Category	Auto-vacuum		Pistol		
		n	(%)	n	(%)	p
Center	HC/FM/UFG	131	(73)	125	(73)	0.9
	HAJ/ACCG	49	(27)	46	(27)	
Age (years)	Mean	38.1		38.4		0.9
Age (years)	Up to 20	24	(13)	27	(16)	0.8
	21-40	86	(48)	77	(45)	
	41-60	54	(30)	54	(31)	
	61 or older	16	(9)	13	(8)	
Tumor size (mm)	Mean	27.3		27.1		0.9
Tumor size (mm)	Up to 20	98	(54)	95	(56)	0.9
	21-40	57	(32)	53	(31)	
	> 40	25	(14)	23	(13)	
Clinical stage*	I	6	(3)	13	(8)	0.3
	II	24	(13)	27	(16)	
	III	19	(11)	17	(10)	
	Benign	130	(73)	114	(66)	

* *For one patient there was no information on this variable; The chi-squared test was used to analyze the qualitative variables and Student's t test was used to analyze the mean.*

*HC/FM/UFG = Hospital das Clfnicas, Faculdade de Medicina da Universidade Federal de Goias; HAJ/ACCG = Hospital Araujo Jorge, Associaqao de Combate ao Cancer em Goias.*

Table 3 illustrates the cytological results for the remaining 351 cases. The percentage of cases with insufficient material was 16% in the group that was aspirated with the auto-vacuum device and 22% in the group in which the pistol-grip mechanical syringe holder was used. Comparison of the rates of insufficient material, using the chi-squared test, showed that there was no statistically significant difference between the two collection methods (p = 0.18).

**Table 3.** Distribution of the cytological diagnosis in 351 patients submitted to fine-needle aspiration biopsy according to the device used in aspiration: auto-vacuum or pistol-grip

Category	Auto-vacuum		Pistol	
	N	(%)	n	(%)
Malignant	24	(14)	25	(15)
Suspicious	8	(4)	7	(4)
Benign	119	(66)	101	(59)
Insufficient material	29	(16)	38	(22)
**TOTAL**	**180**	**(100)**	**171**	**(100)**

*Chi-squared test for insufficient material = 1.7 (p = 0.18).*

With regard to the cellularity of the aspirates, it could be seen that there were greater numbers of glass slides with abundant and moderate material in the group in which the auto-vacuum device had been used. However, this difference was not statistically significant (p = 0.4), as can be seen in Table 4A. It was found that cell distortion artifacts appeared in few cases and were very similar in quantity for the two groups (Table 4B). There was no statistical difference between the groups (p = 0.7). There was a high incidence of hemorrhagic aspirates in both groups. However, these artifacts interfered with the cytological study in only 3% of the punctured smears generated by the auto-vacuum device and in 5% of those harvested using the pistol-grip mechanical syringe holder. There was no difference in hemorrhagic background between the two groups (Table 4C). The accuracy of the FNAB according to the device used is presented in Table 5.

**Table 4.** Distribution of the aspirates, in terms of cellularity of the samples, cell distortion, and hemorrhage, according to the device used in the fine-needle aspiration of 351 breast tumors: auto-vacuum or pistol-grip

(A) Cellularity of the aspirates	Auto-vacuum		Pistol		
	n	(%)	n	(%)	p
Abundant	61	(34)	50	(29)	0.4
Moderate	57	(32)	51	(30)	
Scarce/absent	62	(34)	70	(41)	
**(B) Cell distortion**	**Auto-vacuum**		**Pistol**		
	**n**	**(%)**	**n**	**(%)**	**p**
Present	31	(17)	26	(15)	0.7
Absent	149	(83)	145	(85)	
**(C) Hemorrhagic background**	**Auto-vacuum**		**Pistol**		
	**n**	**(%)**	**n**	**(%)**	**p**
Present, interfering with cytological analysis	5	(3)	9	(5)	0.27
Present, not interfering with cytological analysis	58	(32)	45	(26)	
Absent	117	(65)	117	(69)	

**Table 5.** Accuracy of fine-needle aspiration biopsy according to the device used: autovacuum or pistol-grip

Test	Auto-vacuum	Pistol	p
	%	%
Sensitivity	88	86	0.8
Specificity	99	100	0.3
Positive predictive value	96	100	0.3
Negative predictive value	96	95	0.7
**Total accuracy**	**96**	**96**	**1**

*Patients with suspicious or insufficient samples (nondiagnostic samples) were not included in this analysis, which comprised 273 patients (135 in the auto-vacuum group and 138 in the pistol group). The histology of the specimen was considered to be the gold standard.*

## DISCUSSION

With the use of pistol-grip mechanical syringe holders, the technique of fine-needle aspiration biopsy has become much simpler. This has allowed the operator to concentrate more adequately on the task at hand and focus his/her attention on the direction the needle is taking. The result is more uniform aspirates and more adequate harvesting of the material.

The disadvantages of using the pistol-grip mechanical syringe holder are its relatively large size and weight, when fully assembled. These cause the operator to lose his/her sense of touch to a certain extent, and it is possible that while performing the aspirate, the operator may miss the sensation of the needle passing through the tumor. It must be kept in mind that this sensitivity is important as a complementary form of evaluating the test and must always be taken into consideration, along with the clinical test, in those cases where there is a suspicion of malignancy.^4,12^ Another negative aspect has to do with the fact that, because of its size, the patient may become tense and less willing to collaborate, upon seeing the device fully assembled.

In order to facilitate the puncture, other devices that can be held like pens have been introduced by different researchers.^6,13-15^ The advantages of these devices, relative to the pistol-grip mechanical syringe holders, have recently been described and include the smaller distance between the operator's hand and the lesions, greater sense of touch and constant aspiration. These factors, according to the authors, would facilitate harvesting the material.^15^

However, the literature on these new methods does not establish adequate comparison between these and other techniques that have already become established through usage (e.g. free hand or pistol-grip mechanical syringe holder). The results from the present study enable the establishment of direct and adequate comparison between a prehensile grip device and the classic pistol-grip mechanical syringe holder for fine-needle aspiration.

Care was also taken to ensure the absence of any bias with respect to the tumor-related factors and with respect to the subjectivity of the cytological evaluation. Therefore, a single cytologist was made responsible for analyzing all of the study material. It was also important for the cytopathologist to be blind with respect to what type of device had been used in each case, in order to prevent any possible influence from one method or the other. For this reason, the cytological report contained few clinical data, and most importantly, it did not state which method was used for sampling.

According to data presented by other authors, the rate of insufficient material ranges from 3% to 41%.^16,17^ However, most papers report that percentages ranging from 6% to 20% are acceptable.^18–22^ In the present study, a high rate of insufficient material was found (19%). In spite of the fact that the pistol-grip mechanical syringe holder yielded a higher number of insufficient aspirates (22% versus 16%), this difference did not reach statistical significance, thus showing that this rate was not influenced by the method used for the puncture.

We believe that this high rate of insufficient material may be due to two factors. First, the fine-needle aspiration biopsy was performed by professionals who were in training and still going through their learning curve in terms of this technique. The second reason is related to the large number of benign lesions, including 44 cases of fibrocystic disorders and 28 cases of other benign lesions (lipomas, hamartomas, etc.), plus 85 cases in which the lesion was not removed. These 157 cases totaled 44.7% of all the aspirates included in the study, and represent changes that, in themselves, have a low rate of cellularity.

In order to verify that hypothesis, we used the chi-squared test to compare the cytological findings and types of lesion that were resected. We were able to confirm other authors’ data and to partly justify why we had found a high rate of insufficient material: 12% of the cases with insufficient material in the sample presented fibroadenomas, 20% had carcinomas and 31% had fibrocystic disorders (X^2^ = 10.3; p = 0.005).

Since we believed that the variation in aspirate intensity could interfere by causing hemorrhage during the puncture, the background hemorrhage was studied. Categories were created to help in the assessment, since there was a lack of such data in the literature. The results presented here show that there was background hemorrhage in 35% of the aspirates performed using the auto-vacuum device and in 31% of the aspirates performed using the pistol-grip mechanical syringe holder. Fortunately, the background hemorrhage interfered with the analysis of the aspirate in only a small proportion of the cases: 3% and 5%, for the auto-vacuum device and pistol-grip holder groups respectively. Such similarity of results demonstrates that the type of device had no influence on the severity of the background hemorrhage.

Cell distortion was another factor that might have an influence during the puncture. This variable was designated simply as present or absent, because of the lack of data in the literature. In spite of obtaining a considerable number of aspirates with cell distortion (17% and 15%, for the auto-vacuum and pistol-grip holder groups respectively), the puncture methods did not influence the cellularity of the samples.

Since many operators use fine-needle aspiration biopsy guided by ultrasound, the auto-vacuum device could possibly be very useful in these cases, because the ultrasound technician would not need to create a negative pressure, but simply place the needle inside the lesion and monitor the puncture through the sound transducer.

## CONCLUSION

The results obtained from the two fine-needle aspiration biopsy methods were equivalent. Therefore, the auto-vacuum device is a good option for obtaining aspirates for cytology. The present study not only validates this method but also gives the professionals who deal with breast diseases a further option: one that has been tested, proved to be safe and well within this reach.
